# Both the anti- and pro-apoptotic functions of villin regulate cell turnover and intestinal homeostasis

**DOI:** 10.1038/srep35491

**Published:** 2016-10-21

**Authors:** Yaohong Wang, Sudeep P. George, Swati Roy, Eric Pham, Amin Esmaeilniakooshkghazi, Seema Khurana

**Affiliations:** 1 Department of Physiology, University of Tennessee Health Science Center, Memphis, TN 38163, USA; 2 Department of Biology and Biochemistry, University of Houston, Houston TX 77204, USA; 3 Baylor College of Medicine, Houston TX 77030, USA.

## Abstract

In the small intestine, epithelial cells are derived from stem cells in the crypts, migrate up the villus as they differentiate and are ultimately shed from the villus tips. This process of proliferation and shedding is tightly regulated to maintain the intestinal architecture and tissue homeostasis. Apoptosis regulates both the number of stem cells in the crypts as well as the sloughing of cells from the villus tips. Previously, we have shown that villin, an epithelial cell-specific actin-binding protein functions as an anti-apoptotic protein in the gastrointestinal epithelium. The expression of villin is highest in the apoptosis-resistant villus cells and lowest in the apoptosis-sensitive crypts. In this study we report that villin is cleaved in the intestinal mucosa to generate a pro-apoptotic fragment that is spatially restricted to the villus tips. This cleaved villin fragment severs actin in an unregulated fashion to initiate the extrusion and subsequent apoptosis of effete cells from the villus tips. Using villin knockout mice, we validate the physiological role of villin in apoptosis and cell extrusion from the gastrointestinal epithelium. Our study also highlights the potential role of villin’s pro-apoptotic function in the pathogenesis of inflammatory bowel disease, ischemia-reperfusion injury, enteroinvasive bacterial and parasitic infections.

The small intestinal (SI) epithelium forms the largest and most significant barrier allowing the selective absorption of nutrients, electrolytes and water while maintaining a strict and effective barrier against intra-luminal toxins, antigens and enteric bacteria. SI epithelial cells are tightly adherent cells attached to each other and the extracellular matrix resulting in an architecture that fulfills the protective barrier function of the gut. This tissue composition in turn is maintained by the stringent regulation of cell number within the epithelium by a process that balances cell proliferation with cell death. In all mammalian small intestine, new epithelial cells are generated by the stem cells of the crypts of Lieberkühn every 2–6 days^1^. These cells differentiate as they migrate up the villi to form a functional epithelium. Finally, loss of senescent epithelial cells occurs in the extrusion zone near the villus tips. This cell loss from the villus tips is compensated by stem cell mitosis within the crypts. Apoptosis is the mechanism by which unwanted cells are eliminated from the epithelium and the process by which the cells are squeezed out of the epithelium is termed cell extrusion. While cell shedding occurs coincident with apoptosis, it is thought that extrusion drives cell death^2^. This is based on the observation that apoptosis is virtually never found at the villus tip even though cells are shed at a rate of 1000 cells/villus per 24 h^3^. No apoptotic response is seen in the post-mitotic villus enterocytes along the crypt-villus axis either^4^. Furthermore, shedding in mice and humans is morphologically similar and has been shown to involve whole-cell extrusion and the shed enterocyte is not associated with lymphocytes or macrophages^5^. So in the gastrointestinal (GI) tissue, the proliferative compartment is restricted to the crypts where the stem cells are located while cell shedding is confined to the villus tips. Additionally, apoptotic bodies are seen only in the crypts while along the length of the villus neither apoptotic bodies nor extruding cells have ever been observed^6^. Intestinal epithelial cell shedding remains a poorly understood phenomenon. For instance, despite the high rates of intestinal epithelial cell loss from the villi, shedding events are rarely observed in fixed specimens. Moreover, although cell shedding has been quantified in multiple studies, little is known about the molecular mechanism(s) that mediate cell shedding from the villus tips. Similarly, while much is known about cell proliferation in the gut, much less is understood about apoptosis in the GI tract.

Pathological epithelial cell shedding is associated with several disease states including inflammatory bowel disease (IBD), bacterial infections such as *Salmonella typhimurium*, *Cryptosporidium parvum* and *Bacteroides fragilis*; exposure to bacterial lipopolysaccharide (LPS), tumor necrosis factor (TNF), indomethacin and Toll-like receptor 3 agonists; ischemia and ischemia-reperfusion injury, burn injury, trauma, increased lymphatic pressure; and in cocaine- or atropine-induced villus contraction^7^,^8^,^9^,^10^,^11^,^12^,^13^,^14^,^15^,^16^,^17^,^18^. It is also proposed that the host cells induce enterocyte shedding in response to pathogenic bacteria as an antimicrobial defense mechanism to restrict bacterial replication^19^,^20^. In this case also, extrusion drives pyroptosis. Excessive enterocyte shedding has also been linked to increased intestinal permeability and clinical diarrhea in patients with IBD and has been used to predict disease relapse in these patients^7^,^9^. The current thinking is that abnormally excessive shedding can alter the intestines response to luminal antigens resulting in pathological immune and inflammatory response such as in IBD^21^.

It has been shown that one of the first events in cells destined to undergo extrusion is redistribution of actin and actin associated proteins^22^,^23^. Villin is an actin binding protein that is widely expressed from slime molds to humans and is found in most significant amounts in GI epithelial cells. We have previously reported that villin functions as an anti-apoptotic protein in the GI tract by maintaining tightly regulated actin dynamics^24^. Furthermore we reported that the expression levels of villin along the crypt-villus axis (lowest in crypts and highest along the length of the villus) correlate with the susceptibility of crypt cells and the extreme resistance of post-mitotic villus cells to apoptosis^24^,^25^. In this study, we demonstrate for the first time, that villin is cleaved in the mouse epithelium such that the cleaved NH_2_-terminal fragment of villin is pro-apoptotic and is expressed exclusively in the epithelial cells near the villus tips. Together with our previous study, we demonstrate here that while the anti-apoptotic function of full-length villin is physiologically important for maintaining the resistance to apoptosis in the post-mitotic villus cells, the cleaved villin’s pro-apoptotic function is required for intestinal cell turnover and regulates the sloughing of cells from the villus tips. In this study, we provide a molecular and physiological basis for villin as both an inhibitor but also an effector of apoptosis. Additionally, we describe a molecular mechanism for cell extrusion that is regulated by an epithelial cell specific actin regulatory protein, villin. Since epithelial cell shedding in humans is morphologically similar to that observed in mice, our study also provides novel insight into the physiological function of villin in the human GI tract^26^.

## Experimental Procedures

### Cell lines and Cell Culture

All methods were performed in accordance with the relevant guidelines and regulations. MDCK Tet-Off cells expressing super enhanced yellow fluorescent protein (SEYFP)-tagged wild-type (VIL/WT) or mutant villin (VIL/S4-S6) were transfected by using Lipofectamine 2000. Transfected cells were cultured in Dulbecco’s modified Eagle’s medium containing 100 μg/ml G418 sulfate, 100 μg/ml hygromycin B, and 10% fetal bovine serum. Cells expressing cerulean tagged VIL/S4-S6 were cultured in Dulbecco’s modified Eagle’s medium containing 100 μg/ml zeocin and 10% fetal bovine serum. To repress the expression of villin gene in MDCK Tet-Off transfected cells, cells were cultured in the presence of 10 ng/ml doxycycline as described previously^27^.

### Antibodies and Reagents

Monoclonal antibodies against NH_2_ and COOH-termini of villin (V-20 and C-19 respectively) were purchased from Santa Cruz biotechnology and Immunotech; the actin and tubulin antibodies were purchased from BD Transduction laboratories. Antibodies against cleaved caspase-3 were purchased from Cell Signaling.

### Intestinal epithelial culture

Small intestinal crypts were isolated and cultured in Matrigel in the presence of conditioned media containing EGF, R-spondin, N2 supplement, B27 supplement and Noggin to generate epithelial enteroids^28^. Viable enterocytes were imaged as described previously^29^.

### Purification of glutathione S-transferase-tagged recombinant protein

Full-length and truncated villin proteins were cloned in pGEX-2T and were expressed in *E. coli* BL21 cells and purified as described before^30^.

### Cloning of SEYFP-tagged VIL/S4-S6

Cerulean tag and COOH-terminal fragment of villin (S4-S6) were inserted sequentially into the pBudCE4.1 vector. The cerulean tag was amplified, restricted and inserted into the NotI and KpnI site, as described previously^31^. The S4-S6 COOH-terminal fragment of villin was inserted into the XhoI and BstBI sites. Primers used for inserting S4-S6 fragment of villin which contained the Xho1 and BstB1 sties were 5′GATAGCCTCGAGATCGGCCGTCTTTCAG3′ and 5′CCGCTCTTCGAAAAATAGTCCT-TTTTC3′.

### Actin Depolymerization Kinetics

The severing activity of full length (VIL/WT) and truncation mutant (VIL/NT) of villin was determined by analyzing the rate of decrease in fluorescence of pyrene labeled actin as described before^30^. The villin proteins were incubated in the absence or presence of varying concentrations of Ca^2+^ (0–200 μM) and F-actin.

### Villin Knock-out Mice

All experimental protocols were approved by IACUC (institutional animal care and use committee). Villin knockout mice were generated as described previously^24^. Mice were treated with γ-radiation as described previously^25^. Intestinal brush border membranes were isolated from WT littermates as described previously and used to characterize full-length and cleaved villin fractions using Western analysis^32^. Apoptosis was measured in VKO and WT mice by counting TUNEL-positive nuclei, as well as histologically in hematoxylin and eosin stained sections of the small intestine in 100 epithelial cells per high powered filed, and a total of three fields were counted per section of mouse small intestine. TUNEL staining of intestinal cells always overestimates the rate of apoptosis compared to hematoxylin and eosin (H and E) staining. Consequently, H and E stained images together with TUNEL assay were used because TUNEL staining of villi can produce unreliable non-specific binding due to increased sensitivity of villus cells to proteinase K^6^. Enumeration of apoptotic bodies also allowed quantification of apoptotic cells^33^. Apoptosis was also detected in isolated intestinal epithelial cells from WT and VKO mice using an antibody against cleaved caspase-3. For these studies, mice were given a 3% (w/v) solution of DSS for 5 consecutive days as described previously^24^.

### Apoptosis assay

Cells were subjected to apoptotic stress by incubating with camptothecin (CPT) (20 μM, 37 °C, 0–8.5 h) as described previously^24^. Hoechst 33258 (10 ng/ml) was added to the culture medium for 10 min at 37 °C. All images were collected using a Nikon TE2000 fluorescence inverted microscope equipped with a CoolSnap FX charge-coupled device camera (Roper Scientific, Trenton, NJ, USA) and MetaMorph image analysis software 4.01 (Universal Imaging, Dowington, PA, USA). Apoptotic cells were quantified using Guava easyCyte by flow cytometer as described before^25^. Confocal images were obtained using the Olympus Fluoview FV1200 Laser scanning confocal Microscope using a 60X NA1.35 objective.

### Statistical analysis

Statistical analysis was performed using the two-tailed Student’s t test; p was based on upaired samples and unequal variance. The error bars are the measured standard error (S.E.) of mean.

## Results

### Villin is cleaved in response to prolonged apoptotic stimuli

**Figure 1:**
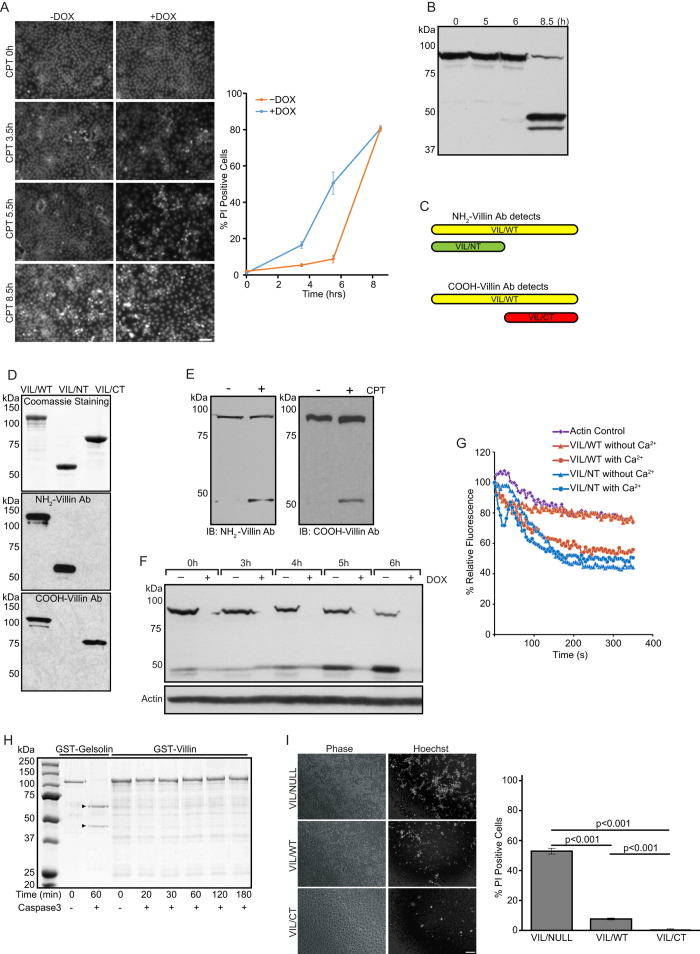
Villin is cleaved during apoptosis to generate a pro- and anti-apoptotic fragment. (**A**) MDCK cells expressing seYFP-tagged full-length villin (VIL/WT) were grown either in the presence (-villin) or absence (+villin) of doxycycline (Dox). Cells were treated with 20 μM CPT for different time intervals (0–8.5 h) and stained with Hoechst 33258. Bar, 50 *μ*m. Cell viability was quantitated using PI staining. This is representative of 8 other experiments with similar data. (**B**) Cells were checked by Western blot for villin. Data are representative of three experiments with similar results. (**C**) Model depicting antibodies used to characterize villin cleavage. (**D**) Specificity of villin antibodies for the NH_2_-terminus and COOH-terminus epitopes of villin was confirmed by Western analysis using recombinant full-length villin (VIL/WT) and truncated (NH_2_-terminus, VIL/NT and; COOH-terminus, VIL/CT) villin proteins. This is a representative of 3 other experiments with similar results. (**E**) Villin cleavage fragments were analyzed in the lysate of CPT treated (20 μM, 8.5 h) MDCK cells expressing VIL/WT. (**F**) Figure shows decrease in full-length and increase in cleaved NH_2_-terminal fragment of villin with time. This is a representative of three other experiments with similar results. (**G**) Actin severing activity of full-length (VIL/WT) and cleaved VIL/NT villin fragment was compared in the presence or absence of calcium. Control represents the polymerization of actin in the absence of villin. This is a representative of three experiments with similar results. (**H**) Recombinant gelsolin or villin proteins were incubated with or without caspase-3 (1.8 ng) for 0–180 min at 37 °C. The cleavage of proteins was analyzed by SDS-PAGE and Gelcode blue staining. Arrowheads indicate the two cleaved fragments of recombinant gelsolin. Data are representative of three experiments with similar results. (**I**) Cells over expressing seYFP-tagged VIL/WT and VIL/CT proteins were treated with CPT and apoptotic cells were identified in phase contrast images, by Hoechst 33258 staining or using PI stain. Viable cells were quantified using flow cytometry. Bar, 50 μm. The error bars are the measured S.E.

In mouse intestinal epithelium villin functions as an anti-apoptotic protein to regulate GI homeostasis^24^,^25^. Additionally, in response to apoptotic injury, over expression of villin delays apoptosis up to 6 h post-treatment^24^. However, we also noted that prolonged exposure to apoptotic stimuli or injury results in the loss of villin’s protective effect on cell survival (Fig. 1A; at 6 h post treatment, p < 0.01, n = 8). Several actin associated proteins and actin itself are cleaved by proteases late during apoptosis^34^,^35^. We hypothesized that villin could be cleaved during prolonged apoptotic injury, rendering the protein non-functional. To test this, we subjected MDCK cells expressing full-length villin to CPT treatment and noted that at 8.5 h post-treatment, the majority of the villin protein is cleaved to generate two fragments of approximately equal size (between 40–45 kDa; Fig. 1B).

### Villin is cleaved into pro- and anti-apoptotic fragments

**Figure 2:**
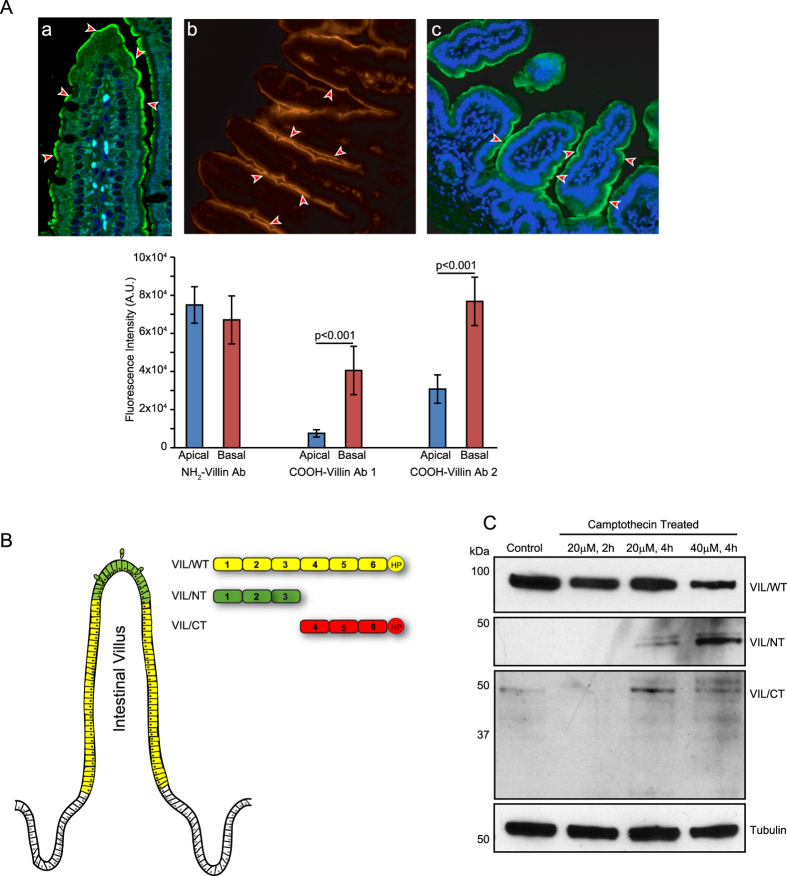
Cleaved NH_2_-terminal villin fragment is generated near villus tips. (**A**) Immunohistochemistry of ileal tissue from WT littermates with NH_2_-terminal villin detecting antibody, shows apical staining of epithelial cells along the entire length of the crypt-villus axis (a), while a significantly reduced staining was observed at the villus tip with two antibodies detecting specifically, the COOH-terminus of villin (b,c). Villin staining is shown in red or green while nuclear staining is shown in blue (DAPI). This is a representative of four other experiments with similar results. (**B**) Schematic diagram showing that the villus tip predominantly contains the pro-apoptotic NH_2_-terminal villin fragment. (**C**) Western analysis shows a decrease in full-length and COOH-terminal as well as an increase in NH_2_-terminal villin fragment generation in ileal tissue of WT littermates in response to CPT treatment. Data are representative of three experiments with similar results.

To further characterize the cleaved fragments of villin, we utilized antibodies that recognized specifically either the NH_2_-terminal fragment (VIL/NT) or the COOH-terminal fragments (VIL/CT) of villin (Fig. 1C). Full-length recombinant villin protein (VIL/WT) and truncation mutants of villin expressing S1-S3 (a.a. 1–338; VIL/NT) and S4-S6 + headpiece (HP) (a.a. 339–827; VIL/CT) were used to validate the specific epitopes of these antibodies (Fig. 1D). As shown in Fig. 1E, during apoptosis villin is indeed cleaved into an NH_2_-terminal fragment containing the S1-S3 domain and a COOH-terminal fragment containing S4-S6 + HP domains (also see Fig. 2B). This is based on the size of the fragments and the antibody specificity which was characterized using these and additional truncation mutants of villin. Using the antibody that recognizes only the NH_2_-terminal fragment we noted a time dependent decrease in full-length and concomitant increase in the cleaved NH_2_-terminal fragment of villin following CPT treatment of cells (Fig. 1F). Using Hoechst and TUNEL staining, we have previously noted that transient expression of the NH_2_-terminal, S1-S3 fragment of villin functions as a pro-apoptotic fragment^27^. What was not known at that time was, how the S1-S3 or VIL/NT fragment induced apoptosis or whether it had any physiological relevance. Since S1-S3 fragment (VIL/NT) is generated in cells during apoptosis, we elected to characterize the functional properties of this S1-S3 fragment. Villin is an actin severing protein that requires high calcium concentrations (>100 μM) to sever actin^36^. As shown in Fig. 1G, unlike full-length villin, the cleaved S1-S3 (VIL/NT) fragment severs actin in the absence of calcium. This suggests that at physiological calcium concentrations, cleaved villin fragment VIL/NT can disrupt the cell cytoskeleton by depolymerizing actin in an unfettered manner. Such dramatic increase in actin severing in an unregulated fashion would kill the cell and we suggest this explains the pro-apoptotic function of the S1-S3 fragment noted in our previous study^27^. Several actin binding proteins including villin’s closest structural and functional homolog gelsolin, are cleaved by caspase-3^35^. Consequently, we tested the ability of full-length recombinant villin to be cleaved by recombinant caspase-3. We noted that unlike the control protein gelsolin, villin is not cleaved by caspase-3 suggesting that villin can be cleaved in the absence of apoptosis or prior to apoptosis (Fig. 1H). Additionally, we noted that like full-length villin, the cleaved COOH-terminal S4-S6 fraction of villin (VIL/CT) also functions as an anti-apoptotic protein (Fig. 1I). These findings are similar to what has been previously reported for gelsolin^37^. Although both the pro- and anti-apoptotic functions of gelsolin have been studies, the biological relevance of the cleaved gelsolin fragments remains undetermined.

### Villin is cleaved in the GI epithelium as cells migrate along the crypt-villus axis

To determine the physiological relevance of villin’s cleavage in the GI tissue, we characterized the cleavage of villin *in vivo* in the SI of C57BL/6J mice. Antibodies that specifically recognize the COOH- and NH_2_-terminal fragments of villin were used for these studies. As shown in Fig. 2A(a) a strong expression of the NH_2_-terminal fragment of villin restricted to the apical surface of enterocytes was noted all along the length of the villus including the villus tips. In published literature, most villin antibodies directed to an epitope in the NH_2_-terminal region demonstrate a similar distribution of villin^38^. Since the antibody recognizes both the full-length and only the NH_2_-terminal fragment, this suggested to us that either the full-length or the cleaved NH_2_-terminal fragment is expressed throughout the villus length including the villus tips. However, we noted that while the COOH-terminal fragment of villin is expressed along the sides of the villi, its expression is significantly lower and almost absent from the villus tips of the SI (Fig. 2A(b)). To validate this finding, a third antibody raised against the villin headpiece (HP) was used. This COOH-terminal directed antibody likewise demonstrated the absence of either the full-length or the cleaved COOH-terminal fragment of villin at the villus tips (Fig. 2A(c)). Together these data reveal that in the mouse epithelium, villin is cleaved near the villus tips to generate the pro-apoptotic NH_2_-terminal fragment of villin. The use of two different antibodies that recognize different epitopes in the COOH-terminus ruled out any effects that may occur as a consequence of antigen masking. As noted in Fig. 2B, our data demonstrates the predominant expression of full-length villin in the villi of WT mice and an increase in the cleaved NH_2_-terminal fragment near the villus tips. It is worth noting that a similar decrease in the expression of full-length villin in cells near the villus tips has been reported previously although the relevance of this observation was not discussed^38^. Isolating intestinal brush border membranes from mice and using Western analysis, we confirmed the expression of both the full-length and the cleaved NH_2_-terminal fragment of villin in the mouse SI epithelium (Fig. 2C). Similar to data obtained in cells (see Fig. 1F) we noted a time dependent decrease in full-length and a concomitant increase in cleaved NH_2_-terminal fragment of villin following CPT treatment of SI of WT mice (Fig. 2C). Likewise we noted a significant decrease in the level of the cleaved anti-apoptotic COOH-terminal fragment of villin following CPT treatment in SI of WT mice as a consequence of protein degradation.

### Cleaved pro-apoptotic fragment of villin regulates cell extrusion from villus tips

**Figure 3:**
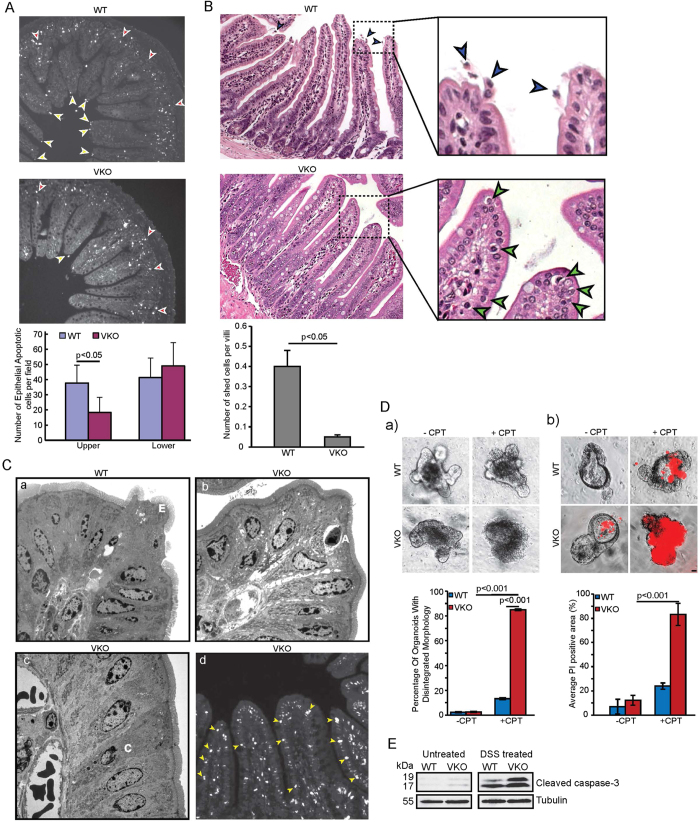
VKO mice have lower number of extruding cells at villus tip. (**A**) Stimulated apoptosis in response to 8Gy radiation was measured in VKO and WT littermates. Apoptotic cells were defined using TUNEL assay. A significant decrease in the number of apoptotic cells at the villus tips was noted in VKO mice compared to their WT littermates (p < 0.05, n = 6). Arrowheads indicate apoptotic cells identified by TUNEL assay in the crypts (red) and villus tips (yellow). (**B**) Histological examination of H and E-stained ileum from WT and VKO mice for extrusion showed extrusion in WT mice (arrowheads) but not VKO mice. VKO mice lack cell extrusion from villus tips but show apoptotic bodies along the crypt-villus axis (arrowhead). WT mice show cell extrusion from villus tips (arrowheads). (**C**) Transmission electron microscopy shows (a) extruding cells (E) in SI of WT littermates while (b) VKO mice show apoptotic bodies (A) and (c) dying cells with crescent nuclei (C). Data are from one representative experiment typical of eight other with similar results. (d) TUNEL assay shows apoptotic cells along the length of the villi (arrowheads) of VKO mice. Data are from one representative experiment typical of eight other with similar results. (**D**) Enteroids from VKO and WT littermate show the apoptotic response of the small intestinal epithelium to CPT treatment. Deletion of full-length villin (VKO) increases apoptotic cells in intestinal epithelium in response to CPT treatment (10 μM, 3.5 h) compared to WT mice. (a) Shows morphological changes in enteroids following CPT treatment; (b) shows changes in PI staining scored using fluorescence microscopy. The error bars are the measured S.E. Bar, 20 μM. (**E**) Western analysis of cleaved caspase-3 in WT and VKO mice untreated or treated with 3% Dextran sodium sulfate (DSS) for 5 days as described in the Methods section. Western blot with anti-tubulin antibody was performed in parallel as a quantitative control. Data are representative of three experiments with similar results.

In the normal adult SI a very low frequency of apoptosis is seen in the crypts of both mice and humans, approximately one apoptotic cell is seen in every fifth histological longitudinal crypt section^39^. Furthermore, this spontaneous apoptosis is never detected in the villus cells^6^. Since “spontaneous” apoptosis occurs at such low almost undetectable rates in the SI, we subjected mice to 8 Gy radiation or DSS-induced injury to measure “stimulated” apoptosis using TUNEL assay or cleaved caspase-3 antibody essentially as described before^24^,^25^. For these studies both villin knockout (VKO) mice and their WT littermates were subjected to 8 Gy radiation and apoptotic cells analyzed 4h post-treatment. It is worth noting that radiation increases the apoptotic bodies in the crypts but the length of the villus in a normal mouse remains resistant to apoptosis despite similar exposure to radiation^40^,^41^. With that in mind, we noted that in WT mice, apoptotic cells were increased near the base in the crypts (red arrowheads) in response to radiation (Fig. 3A). Radiation also resulted in the appearance of apoptotic cells at the villus tips of WT mice but none were seen along the length of the villus (Fig. 3A; yellow arrowheads). The number of apoptotic cells in the crypts and at the villus tips in response to radiation in WT mice is comparable to what has been reported previously in similar studies^6^,^42^. More surprisingly, we noted a significant decrease in the number of apoptotic cells at the villus tips of the VKO mice (Fig. 3A). The apoptotic cells were confirmed using both TUNEL and H and E staining for reasons described in previous studies^6^. Examination of untreated SI using H and E staining further highlighted morphological difference between the SI of WT and VKO mice. Cell extrusion was seen, as expected, from the villus tips of WT mice but surprisingly no cell extrusion could be documented from the villus tips of the VKO mice (Fig. 3B; blue arrowheads). Instead, we noted the presence of apoptotic bodies at the villus tips and along the length and sides of the villi of VKO mice (Fig. 3B green arrowheads). Apoptotic bodies were seen as rounded cells with smooth membrane bound body containing condensed chromatin. Since the villus cells are post-mitotic cells, these structures were definitively identified as apoptotic bodies. To further corroborate this observation we used transmission electron microscopy (TEM) that clearly showed degenerating, extruding cells (E) with characteristic funnel-like structure from the SI of wild-type mice but not VKO mice (Fig. 3C). Non-apoptotic characteristics along with swollen mitochondria and vacuoles were seen in these extruding cells consistent with previous descriptions of extruding cells. In contrast the VKO mice displayed rather typical apoptotic bodies (A) at the villus tips with characteristic well defined halo and condensation and margination of the chromatin. Dying cells with crescent nuclei (C) were also seen by TEM in SI of the VKO mice. It may be noted that similar apoptotic profiles and morphological characteristics of apoptotic bodies have been described but in the crypts and are never associated with the villi^43^. It is also well recognized that while apoptotic bodies are retained within the epithelial monolayer of the crypts, they are never retained within the villi of either the unstressed or stressed intestine^5^,^44^. Using TUNEL assay, apoptotic cells (yellow arrowheads; Fig. 3C(d)) were recorded along the length of the villi of VKO mice. To substantiate these findings, we elected to use primary cells or enteroids derived from WT and VKO mice which demonstrate *in situ* that deletion of villin results in a significant decrease in the number of viable cells in response to apoptotic injury (Fig. 3D). Similar studies could not be done using VKO enteroids to express VIL/NT since this fragment is pro-apoptotic and renders the enteroids unviable, which is similar to our observation made with villin S1-S3 fragment in epithelial cell lines^27^. To validate these findings we also treated VKO and WT mice with 3% DSS to induce apoptosis essentially as described by us previously (Fig. 3E)^24^. As expected, following DSS treatment for 5 days, VKO mice have more apoptotic cells compared to the WT littermates. Apoptosis was measured using a cleaved caspase-3 antibody.

## Discussion

Together with our previous findings, data shown here demonstrate that while full-length villin functions as an anti-apoptotic protein rendering the post-mitotic villus epithelial cells resistant to cell death, the cleaved NH_2_-terminal fragment of villin regulates epithelial cell extrusion from the villus tips by inducing apoptosis^24^,^25^. Our study demonstrates that the expression of full-length and cleaved villin fragment correlates with the cell’s positional status and ability to resist or undergo apoptosis. Thus, both the anti- and pro-apoptotic functions of villin appear to be physiologically relevant for the maintenance of intestinal morphology and homeostasis. We note that in *Drosophila melanogaster* the villin orthologue quail induces apoptosis to produce viable oocytes^45^. This suggests that like its anti-apoptotic function, villin’s pro-apoptotic function may also be a conserved feature of multicellular organisms.

In this study, we identify for the first time a role for villin in the regulation of epithelial cell extrusion. Data shown here confirm that by disassembling actin under physiological calcium concentrations, the cleaved villin fragment can induce cell extrusion from the villus tips. The villus length/height is not significantly altered between the VKO mice and their WT littermates. Therefore, despite the lack of cell extrusion from the villus tips, the intestinal morphology appears reasonably normal in the VKO mice. The deletion of villin inhibits extrusion, but genetic deletion of villin also induces apoptosis in the otherwise extremely resistant villus epithelial cells. More remarkably, we now see the presence of apoptotic bodies all along the crypt-villus axis including the villus tips. The presence of apoptotic bodies in the villi of VKO mice also highlights the change in the molecular mechanism used to remove dead cells from VKO mouse SI compared to their WT littermates. In the normal gut, there is a differences in the mechanisms used by crypt and the villi to remove dead cells namely by *in situ* phagocytosis in crypts *versus* extrusion in villi, respectively. In the absence of villin this mechanism to clear dead cells appears modified. Apoptotic bodies now appear in the villi similar to the crypt suggesting a switch from extrusion to phagocytosis to clear senescent cells from SI of VKO mice. The data show no significant difference in cell turnover or rate of removal of senescent cells. It is known that unlike mouse and human gut, in reindeer and seal gut, senescent enterocytes can be removed both by extrusion as well as by phagocytosis by underlying macrophages^46^. This implies that the molecular machinery for both types of cell removal exist in the mammalian villus cells. We suggest that such a switch in the molecular machinery could be used under potential stress or disease conditions where villin expression is either lost or villin cleavage is compromised.

**Figure 4:**
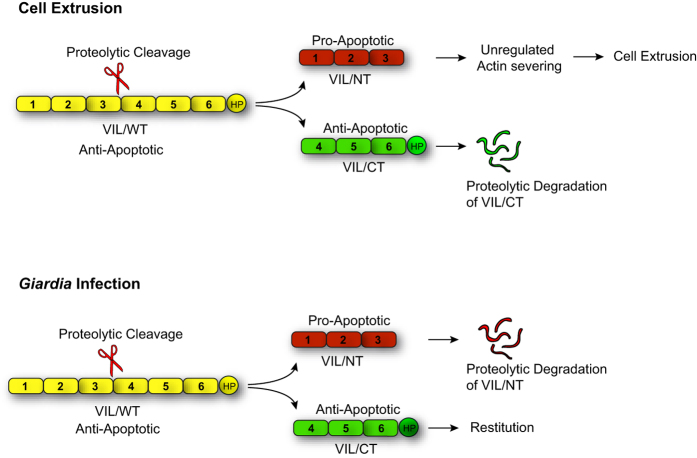
Model showing villin cleavage in physiology and pathophysiology. Model shows the proteolytic cleavage of full-length villin which generates a pro-apoptotic NH_2_-terminal fragment that regulates cell extrusion from villus tips. In *Giardia* infection, proteolytic cleavage generates an anti-apoptotic COOH-terminal fragment that regulates restitution.

In the intestine, defective apical extrusion signaling has been shown to contribute to more aggressive tumor hallmarks^47^. Since villin expression is frequently lost in poorly differentiated colon cancers, we suggest that in such tumors the elimination of transformed cells may be prevented in the absence of villin which could enable tumor cells to survive, hang around longer and ultimately initiate metastasis^48^. Several enteric pathogens (including *Salmonella, V. parahemolyticus*, EHEC and *Entamoeba histolytica*) hijack cell extrusion to invade the gut epithelium^10^. Pathogen-induced cell extrusion is thought to restrict the pathogen replication and colonization. It may be noted that villin cleavage is frequently associated with such enteric invasions^49^. So the cleavage of villin and its pro-apoptotic function may be relevant not only to constitutive cell extrusion at the villus tips but may also play a significant role in pathophysiological conditions where it may serve to reduce the injurious potential of the insult or promote metastasis (Fig. 4). While our study highlights the role of the pro-apoptotic fragment in GI homeostasis, we suggest that the COOH-terminal fragment and its anti-apoptotic function may also have a role *in vivo* such as in restitution and repair of the epithelium. Increased villin proteolysis following intestinal infection with the parasite *Giardia duodenalis* results in the accumulation of the COOH-terminal fragment instead of the NH_2_-terminal fragment^50^. This has been thought to contribute to the regeneration of tissue homeostasis following *Giardia* infection. We suggest that the anti-apoptotic function of the COOH-terminal fragment may play a role in wound repair and re-epithelialization of the gut following such enteric infections.

The closest homolog of villin, gelsolin is cleaved by caspase-3 to generate an NH_2_-terminal pro- and a COOH- terminal anti-apoptotic fragment^35^. Studies comparing substrates of different caspases have demonstrated that the cleavage sites of all caspases share a general motif^51^. Additionally, promiscuity for different cleavage motifs have also indicated that caspase-3 cleaves most substrates more efficiently than any other caspase^51^. Taking this in account, we analyzed the cleavage of villin by caspase-3. The fact that villin was determined to not be a substrate of caspase-3 indicates that villin cleavage could precede apoptosis, this is consistent with the idea that villus cells acquire apoptotic morphology only after they have been shed into the lumen^4^. Cell extrusion in the absence of caspase activation has been described in *C. elegans* suggesting that such mechanisms may not be an exception but the prevailing mechanism in multicellular organisms^52^. In mammalian cells, matrix metalloproteinase (MMP) activity has been shown to regulate apical cell extrusion^53^. Meprins are MMPs that are expressed in very significant amounts in epithelial cells particularly of the GI tract. More importantly, villin is a substrate of meprin^54^. It is likely then that *in vivo* villin is cleaved by meprin to generate the actin-severing NH_2_-terminal fragment to induce cell extrusion from the villus tips.

In summary, our study demonstrates that the expression of full-length and cleaved villin correlates with the cell’s position along the crypt-villus axis and determines the ability of the cells to undergo apoptosis and extrusion. Our study also describes for the first time a physiological molecular mechanism to regulate extrusion from the villus tips. This process appears to be uniquely adapted to the epithelial cells, using an epithelial cell specific actin regulatory protein namely, villin. Further studies will provide greater insight into the complex regulation of cell survival, cell death and cell extrusion that is required for the maintenance of intestinal epithelial architecture and homeostasis.

## Additional Information

**How to cite this article**: Wang, Y. *et al*. Both the anti- and pro-apoptotic functions of villin regulate cell turnover and intestinal homeostasis. *Sci. Rep.* **6**, 35491; doi: 10.1038/srep35491 (2016).
